# Hydatid Cyst in the Hand

**DOI:** 10.1590/0037-8682-0623-2021

**Published:** 2022-04-08

**Authors:** Recep Tekin, Emin Özkul, Sait Anıl Ulus

**Affiliations:** 1 Dicle University, Faculty of Medicine, Department of Infectious Diseases and Clinical Microbiology, Diyarbakir, Turkey.; 2 Dicle University, Faculty of Medicine, Department of Trauma and Orthopedic Surgery, Diyarbakir, Turkey.

A 52-year-old man presented with a 1-year history of limitation in the movement of his left hand and numbness involving the fingers. The physical examination of his hand revealed swelling, 4^th^ and 5^th^ finger loss of feeling, positive Tinel’s sign, and mildly painful movement with minimal limitation, especially in the 4^th^ and 5^th^ fingers. A radiological examination revealed soft tissue swelling of the hand with no bone erosion or calcification (Figure 1). Magnetic resonance imaging of the patient’s hand revealed multiple, round, multivesicular daughter cysts occupying almost the entire volume of the mother cyst confined to the soft tissues at the flexor surface surrounding the all-flexor tendons and muscles of the hand, with no infiltration into the bone or the surrounding neurovascular structures (Figure 2). Following the irrigation of the cystic cavity with a chlorhexidine solution, the cystic mass was wholly excised with its capsule (Figure 3). The multiple daughter cysts were filled with a muddy substance, typical of hydatid disease. Histopathological examination confirmed the diagnosis of hydatid disease. The patient received 6 weeks of oral albendazole at 400 mg daily. Pericystectomy combined with neoadjuvant therapy can help reduce the complications and recurrence in soft tissue hydatid cysts^1^ ^,^ ^2^. Hydatid cyst should be considered for differential diagnosis of soft tissue masses, particularly in patients who live in regions where hydatid cyst is endemic^3^.

**FIGURE 1:**
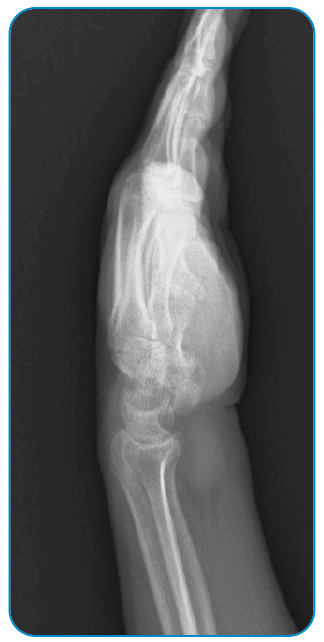
Radiological image showing soft tissue swelling of the hand.

**FIGURE 2:**
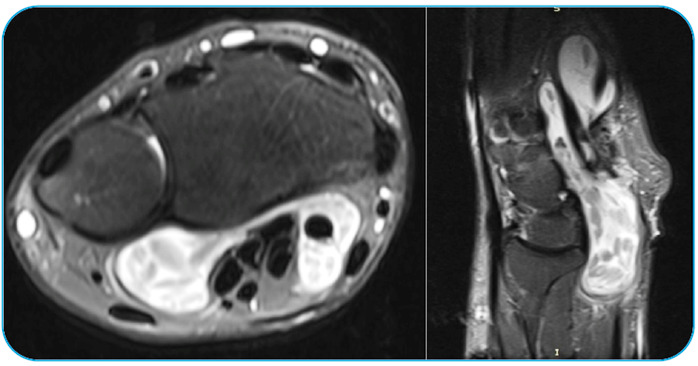
Magnetic resonance imaging of the hand and wrist displaying multiple, round, multivesicular daughter cysts occupying almost the entire volume of the mother cyst confined to the soft tissues at the flexor surface surrounding the all-flexor tendons.

**FIGURE 3:**
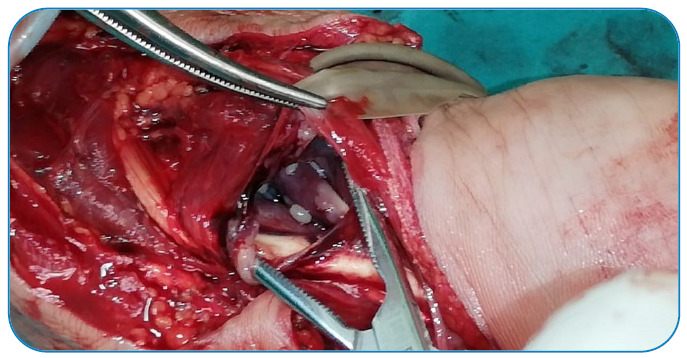
Intraoperative image of multivesicular cysts containing multiple daughter cysts.

## References

[1] Tekin RC, Özkul E, Tekin R (2021). Hydatid cyst in the wrist. Rev Soc Bras Med Trop.

[2] Tekin R, Avci A, Tekin RC, Gem M, Cevik R (2015). Hydatid cysts in muscles: Clinical manifestations, diagnosis, and management of this atypical presentation. Rev Soc Bras Med Trop.

[3] Tekin R, Onat S, Tekin RC (2016). Hydatid cysts in a patient with multiple organ involvement. Rev Soc Bras Med Trop.

